# Podiatrists’ perceptions of Continuing Professional Development (CPD) and the requirement for re-registration

**DOI:** 10.1186/1757-1146-3-S1-O7

**Published:** 2010-12-20

**Authors:** Mark Cole

**Affiliations:** 1 University of Southampton, School of Health Sciences, Southampton, UK

## Introduction

As a part of the NHS modernisation agenda to ensure high quality clinical care, podiatrists are required to demonstrate evidence of continuing professional development to maintain registration. This study aimed to investigate the experiences of podiatrists in response to the CPD process. It explored the effectiveness of the portfolio in recording CPD, resource as a limiting factor in CPD engagement and the linking of competence with re-registration.

## Method

The study was of a qualitative design using purposive sampling to select six key informants. Semi-structured interviews were conducted and data was analysed using themaptic analysis.

## Results

Four main themes emerged from the study**: Called to account -** Perceptions of the CPD process indicating poor comprehension and associated anxiety due to lack of effective communication; **Proving your worth -** Positive views of linking competence with re-registration; **Transparent recording device -**Support for the portfolio but concern for its ability to demonstrate practical competence; **Balancing responsibilities -** Experiences of funding depletion and strategies to maximize free CPD.

## Discussion

This study has been valuable in gaining a deeper understanding of the podiatrist’s perceptions of CPD and may assist in informing future process. Podiatrists were in favour of the new regulations but showed anxiety towards the process due to confusion over the requirements. Support was shown for a link between re-registration and competence to ensure patient safety. Funding deficiencies were a concern resulting in the use of free CPD as part of daily practice.

